# Influence of sports flooring and shoes on impact forces and performance during jump tasks

**DOI:** 10.1371/journal.pone.0186297

**Published:** 2017-10-11

**Authors:** Laurent Malisoux, Paul Gette, Axel Urhausen, Joao Bomfim, Daniel Theisen

**Affiliations:** 1 Sports Medicine Research Laboratory, Luxembourg Institute of Health, Luxembourg, Grand-Duchy of Luxembourg; 2 Sports Clinic, Centre Hospitalier de Luxembourg, Luxembourg, Grand-Duchy of Luxembourg; 3 Mondo Luxembourg SA, Foetz, Grand-Duchy of Luxembourg; Northwestern University, UNITED STATES

## Abstract

We aim to determine the influence of sports floorings and sports shoes on impact mechanics and performance during standardised jump tasks. Twenty-one male volunteers performed ankle jumps (four consecutive maximal bounds with very dynamic ankle movements) and multi-jumps (two consecutive maximal counter-movement jumps) on force plates using minimalist and cushioned shoes under 5 sports flooring (SF) conditions. The shock absorption properties of the SF, defined as the proportion of peak impact force absorbed by the tested flooring when compared with a concrete hard surface, were: SF0 = 0% (no flooring), SF1 = 19%, SF2 = 26%, SF3 = 37% and SF4 = 45%. Shoe and flooring effects were compared using 2x5 repeated-measures ANOVA with post-hoc Bonferroni-corrected comparisons. A significant interaction between SF and shoe conditions was found for VILR only (p = 0.003). In minimalist shoes, SF influenced Vertical Instantaneous Loading Rate (VILR) during ankle jumps (p = 0.006) and multi-jumps (p<0.001), in accordance with shock absorption properties. However, in cushioned shoes, SF influenced VILR during ankle jumps only (p<0.001). Contact Time was the only additional variable affected by SF, but only during multi-jumps in minimalist shoes (p = 0.037). Cushioned shoes induced lower VILR (p<0.001) and lower Contact Time (p≤0.002) during ankle jumps and multi-jumps compared to minimalist shoes. During ankle jumps, cushioned shoes induced greater Peak Vertical Ground Reaction Force (PVGRF, p = 0.002), greater Vertical Average Loading Rate (p<0.001), and lower eccentric (p = 0.008) and concentric (p = 0.004) work. During multi-jumps, PVGRF was lower (p<0.001) and jump height was higher (p<0.001) in cushioned compared to minimalist shoes. In conclusion, cushioning influenced impact forces during standardised jump tasks, whether it was provided by the shoes or the sports flooring. VILR is the variable that was the most affected.

## Introduction

Jumping is an essential athletic task used during many different sporting activities, such as basketball, volleyball or gymnastics. Impact forces experienced during ground contact in landings can reach a magnitude of 3 to 7 times body weight [1]. The rate at which impact forces develop also deserves attention since loading rate has been associated with increased injury risk in running [2, 3]. Indeed, a meta-analysis showed that the loading rate of the impact force is significantly higher among people with stress fractures compared to control groups [4]. It is common belief that impact forces and loading rate are associated with the risk of overuse injuries in sports requiring repetitive jumps [5, 6], however the level of evidence is still weak. To reduce the potential risk of injury associated with high vertical ground reaction forces, different interventions (e.g. cushioned shoes, patellar taping, feedback on landing technique…) have been used to decrease ground reaction forces by altering lower extremity biomechanics during landing [7–9]. The concept of cushioning has been used for several decades in footwear engineering in an attempt to reduce such impacts [10, 11]. However, the results of the many studies focussing on the effects of shoe hardness (or materials) in reducing impact force peaks and loading rate have been to some extent inconclusive [9, 12, 13].

In addition to the sports shoe, sports flooring (SF) represents another material that comes as an interface between the foot and the ground during landing. Multifunctional point elastic SF consist of layers of natural and synthetic rubber and an elastic foam supporting underlay. Shock absorption is assessed with a specific apparatus (termed Artificial Athlete Apparatus) [14], according to a standard method which consists in comparing the tested surface to a completely rigid flooring construction. The method measures how much of the impact force (%) is absorbed by the tested flooring [15]. Although widespread, this test method also generates some criticism for being too simple and not giving enough insight on athlete-flooring interactions under real conditions [16].

Little information is available on the interaction between the athlete’s biomechanics and SF properties. This is surprising, since this interaction might play a role in the athletes’ performance [17], as well as their safety. Additionally, this lack of knowledge represents a barrier to designing the optimal solution regarding safety and performance [14]. Most studies investigating impact attenuation have focused on the role of footwear in shock absorption during running [11, 12, 18], but little is known about the influence of shoes or surfaces on landing activities [9, 16]. Indeed, care must be taken when extrapolating observations from running to other activities, as several studies showed that cushioning effects are task-specific [9, 19].

The aim of this study was to determine the influence of SF, as well as sports shoes, on impact forces and jump performance during standardised jump tasks. Different SF with specific technical properties were compared to identify which are most efficient in lowering impact forces at landing. The primary hypothesis was that the SF with the highest shock absorption properties would display a lower vertical instantaneous loading rate (VILR) as well as a lower peak vertical ground reaction force (PVGRF) compared to the SF with the lowest shock absorption properties.[20] Our secondary hypothesis was that shoe features would also influence impact forces, and therefore, that VILR and PVGRF would be lower in cushioned shoes compared with minimalist shoes. Finally, given the lack of consistency between the few studies that investigated the effect of cushioning on jump performance [17, 21], we hypothesised that jump performance would not be affected by shoe and SF conditions.

## Materials and methods

### Study participants

A convenience sample of 41 physically active and healthy men were contacted in local sports clubs in November 2016. Twenty-one volunteers (age: 26.8±5.7 years, height: 1.85±0.07 m, and body mass—BM: 79.9±10.4 kg) accepted to participate in this cross-sectional study and fulfilled the following inclusion criteria: aged 18 to 50 years, shoe size between 42 and 47, experienced in jumping training through regular practice (at least twice weekly) of sports involving jumping tasks (e.g. volleyball, basketball…), no contraindication to jumping and testing, no history of surgery to the lower limbs or the back region within the previous 12 months or any degenerative conditions, and no use of insoles for physical activity. Among the 41 participants initially contacted, 16 had inappropriate shoe size, 3 eventually declined to participate and 1 incurred an injury before entering the study. The participants were requested not to practice any physical activity the day before the test and to have their last meal at least 2 hours before the beginning of the experiment. The volunteers were fully briefed about the study protocol and provided written informed consent for participation. All procedures had previously been approved by the National Ethics Committee for Research (ref. 201609/03 v1.1).

This cross-sectional study is purely exploratory. Nevertheless, based on studies that investigated the effect of shoe features on impact forces [11, 22], we estimated that a difference (mean ± SD) in PVGRF of 0.20 ± 0.29% BM between shoe or SF conditions would give a statistical power of about 80% if the significance level were set at p = 0.05 and 17 participants were included in the study.

### Experimental tasks

Two jump tasks were investigated separately: ankle jumps and multi-jumps. The ankle jumps task consisted of four consecutive maximal jumps with very dynamic ankle movements and straight knees. The participants were requested to jump as high as possible using primarily their lower legs and ankle muscle power, while reducing the contact time as much as possible. The multi-jumps task consisted of two consecutive maximal counter-movement jumps, each preceded by a knee flexion of about 90°. Both tasks were explained and demonstrated to the participants, and several training trials were allowed before recording. Participants performed as many practice attempts as needed to feel comfortable jumping with the tested shoe conditions for each of the experimental tasks. A jump was considered valid if the participant landed with each foot on a force plate and if the hands remained placed at the hips. Each task was repeated 3 times for each condition (combination of shoe models and SF). The order of the conditions was administered in a random sequence for each participant. A 1 minute rest was foreseen between two consecutive records to avoid fatigue, perform a data check and change the testing conditions. All tests were administered within a single session and preceded by a warm-up run of 5–10 minutes on a treadmill at a self-selected pace.

### Conditions

#### Sports floorings

**Fig 1:**
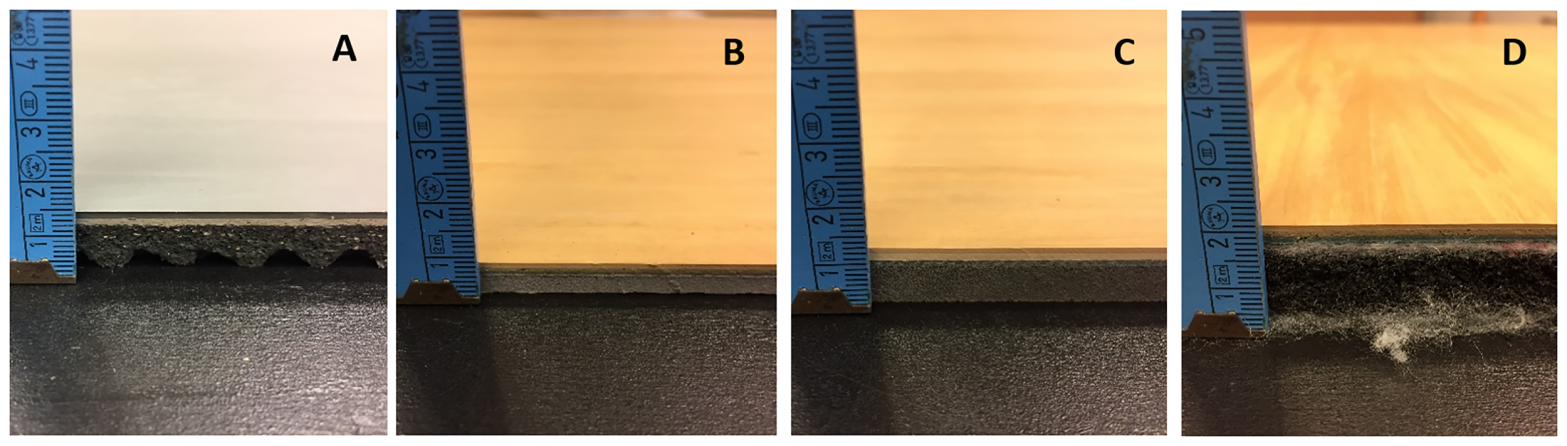
Sports floorings used for the study. A: Sports flooring 1 (SF1 = 14 mm); B: Sports flooring 2 (SF2 = 7.5 mm); C: Sports flooring 3(SF3 = 11 mm); D: Sports flooring 4 (SF4 = 18 mm).

Jumping tasks were performed on four different SF, positioned on top of 2 force plates (see Biomechanical measurements). For each condition, a single sample covered both force plates and was fixed with double-sided adhesive tape. The flooring samples were provided by MONDO (Mondo Luxembourg S.A., Luxembourg) and consisted in layers of rubber and an elastic foam underlay. Their thickness was: SF1 = 14 mm, SF2 = 7.5 mm, SF3 = 11 mm, and SF4 = 18 mm (see Fig 1). Jumps performed on the force plates without any flooring served as the control condition (SF0). Shock absorption properties of the SF (SF1 = 19%, SF2 = 26%, SF3 = 37%, and SF4 = 45%) were provided by Mondo after data treatment, thus ensuring researcher and participant blinding.

#### Sports shoes

**Fig 2:**
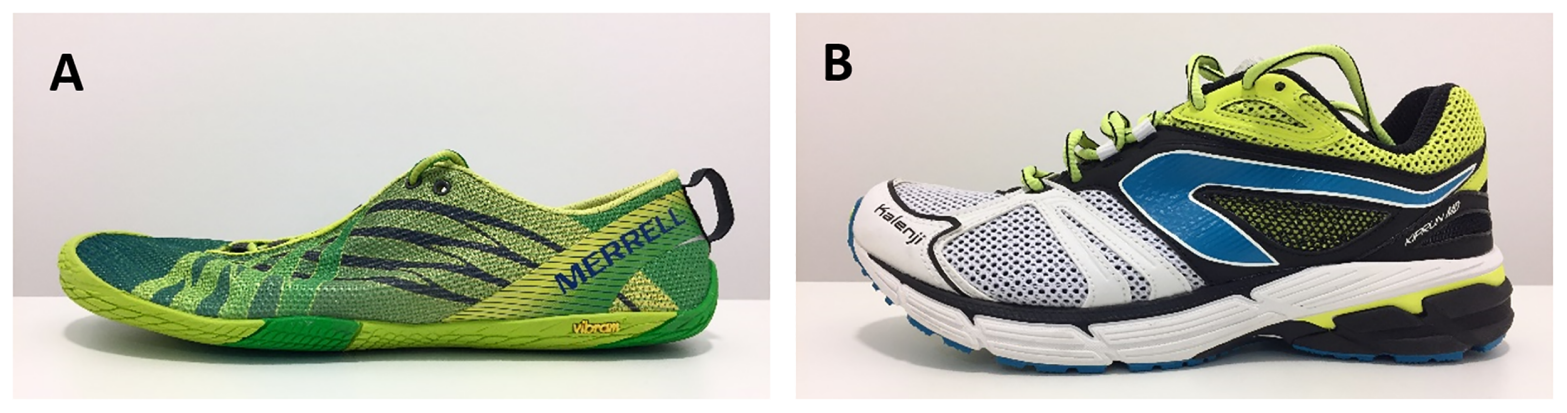
Sports shoes used for the study. A: Minimalist shoes (MIN); B: Cushioned shoes (CUSH).

All jump conditions were performed using two types of running shoes that differed, among other, in cushioning properties (Fig 2). The minimalist shoes (MIN; Merrell Vapor Glove) had a 0 mm heel-to-toe drop, a 5 mm overall stack height, weighed 159 g (size 45) and were very flexible. The standard cushioned running shoes (CUSH; Kalenji Kiprun MD) had greater shock absorption properties and were characterised by 10 mm drop, 26 mm overall stack height, and 320 g shoe mass (size 43).

### Biomechanical measurements

Two Arsalis 3D force plate systems (Arsalis 800x500; Arsalis SPRL, Louvain-la-Neuve, Belgium) were used to capture vertical ground reaction force (F_z_) data at a sampling rate of 1000 kHz. Each analogue output amplifier was configured to the sensitivity of 1.60 mV/N and had the minimal measurable force of 0.25 N. Neither analogue nor digital signal filtering was conducted at any device level to avoid possible signal distortion [23]. Time-synchronised raw signal records of the two force plates were imported into a custom-programmed Matlab graphical user interface (Matlab R2014a, MathWorks, Netherlands). The two force plates were treated as one, which means that the raw signals from both force plates were summed before the calculations. Prior to the experiment, the two force plates were calibrated against weights of known masses.

### Signal processing

**Fig 3:**
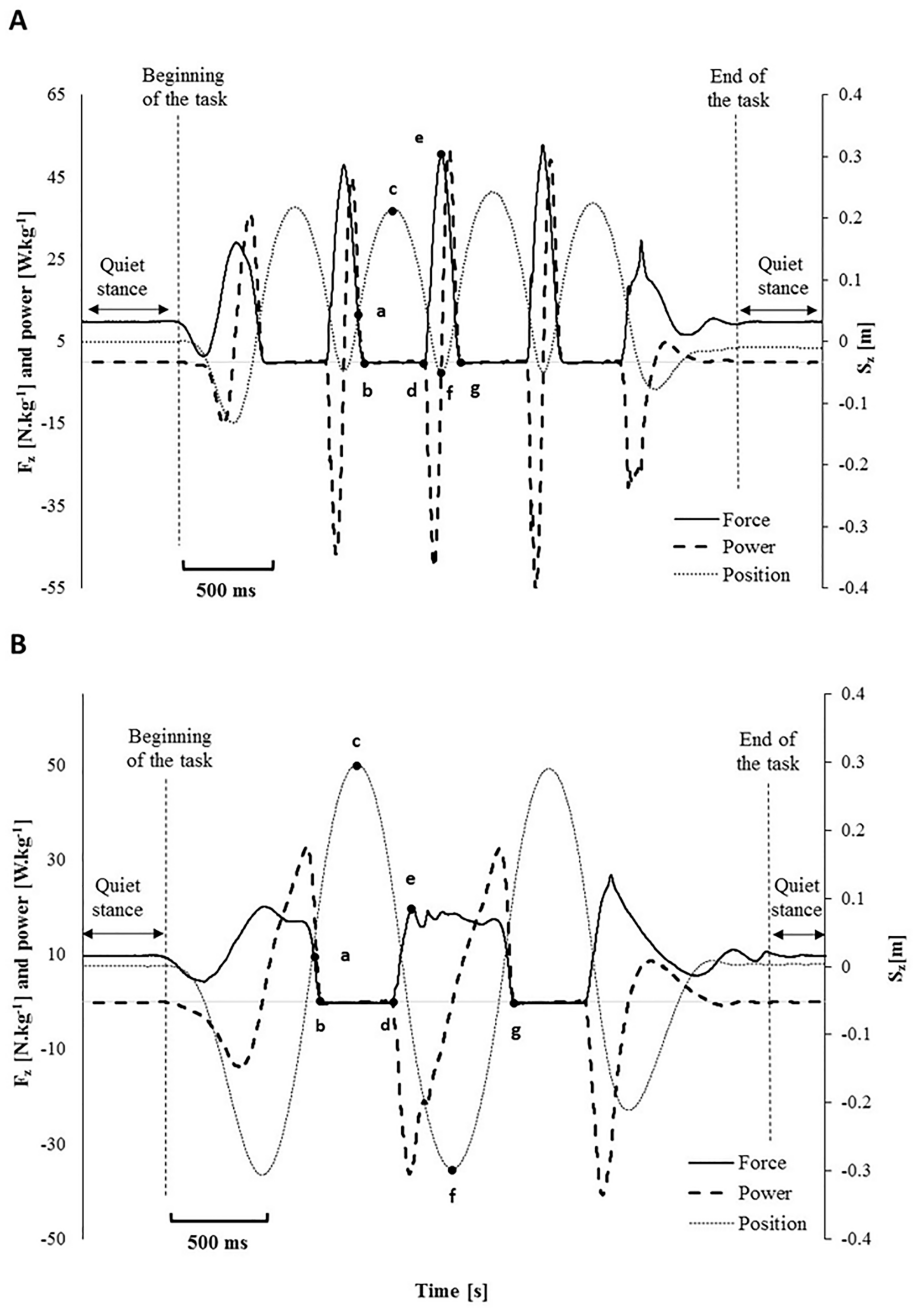
Example of Force (N.kg^-1^), Power (W.kg^-1^) and Position (m) curves for the ankle jumps (A) and multi-jump (B) tasks. a: ground reaction force is equal to body weight propulsion and jumper reaches maximum vertical velocity; b: take-off 1, when ground reaction force < 10 N; c: maximum jump height; d: touch-down; e: peak vertical ground reaction force; f: lower position of COM; g: take-off 2; b-d: Flight time; d-g: contact time.

Participant’s body weight (BW, N) was obtained by the average Fz signal recorded during quiet stance (see Fig 3). The net vertical acceleration (Az, m.s^-2^) of the participant’s centre of mass (COM) was computed as Az = (Fz–body weight)/BM [24]. Net COM vertical velocity (Vz, m.s^-1^) and displacement (Sz, m) were obtained through numerical integration using the trapezoidal rule throughout the record [25]. The scalar product of force and velocity yielded net power, normalised to the participant’s BM (P, in W/kg). Work (W, in J) was calculated by integrating the power curve with respect to time [26]. Negative values represented hereby the net eccentric work (W_ecc_) done to decelerate the COM when landing from a jump, whereas positive values reflect the net concentric work (W_con_) performed to jump up. The transition from the phase of W_ecc_ to the phase of W_con_ during each jump was defined by the corresponding minimum of the COM displacement curve (Fig 3). Touch-down (TD) and take-off (TO) events were determined based on a 10 N threshold from the vertical ground reaction force curve [23]. Flight time (FT, in s) was defined as the time interval between TO and TD, contact time (CT, in s) as the time interval between TD and TO.

To assess the landing impact, several variables were computed in the period from TD to the subsequent lowest COM position. PVGRF (N.kg^-1^) was extracted from the recorded signal. Vertical average loading rate (VALR, N.s^-1^.kg^-1^) was computed as VALR = (Fz_max_—Fz_TD_) / (tFz_max_—t_TD_) and, VILR (N.s^-1^.kg^-1^) as VILR = max (dFz / dt), where (t_TD_ < t < tFz_max_) [27]. Finally, W_ecc_ was calculated between t_TD_ and tFz_max_. Jump task performance was assessed by jump height (JH, m) and W_con_ (J). JH was calculated using the COM vertical displacement curve obtained via double integration [28, 29]. JH value was corrected by the distance of the COM position between quiet stance and TO. W_con_ was calculated between the instant of preceding lowest COM position and the subsequent TO.

### Statistics

The three trials of each subject in each experimental condition were averaged. A 2x5 (shoe x SF) repeated measures analysis of variance (ANOVA) was used to determine the effects of shoes and SF on impact forces and jump performance separately for each jump task. Bonferroni post-hoc tests were used to determine individual significant differences. Stratified analyses according to shoe condition were performed to investigate specific effects of SF in each shoe type, using (1X5) repeated measure ANOVA. The significance level was set at p<0.05. Omega squared (*ω*^2^), defined as the proportion of variance associated with or accounted for by each variable in an ANOVA, was used as measure of effect size [30]. *ω*^2^ values were classified as large (>0.14), medium (0.14–0.06) or small (≤0.06). All analyses were performed using STATA/SE V.14.

## Results

### Effect of sports floorings on PVGR and VILR during ankle jumps

**Fig 4:**
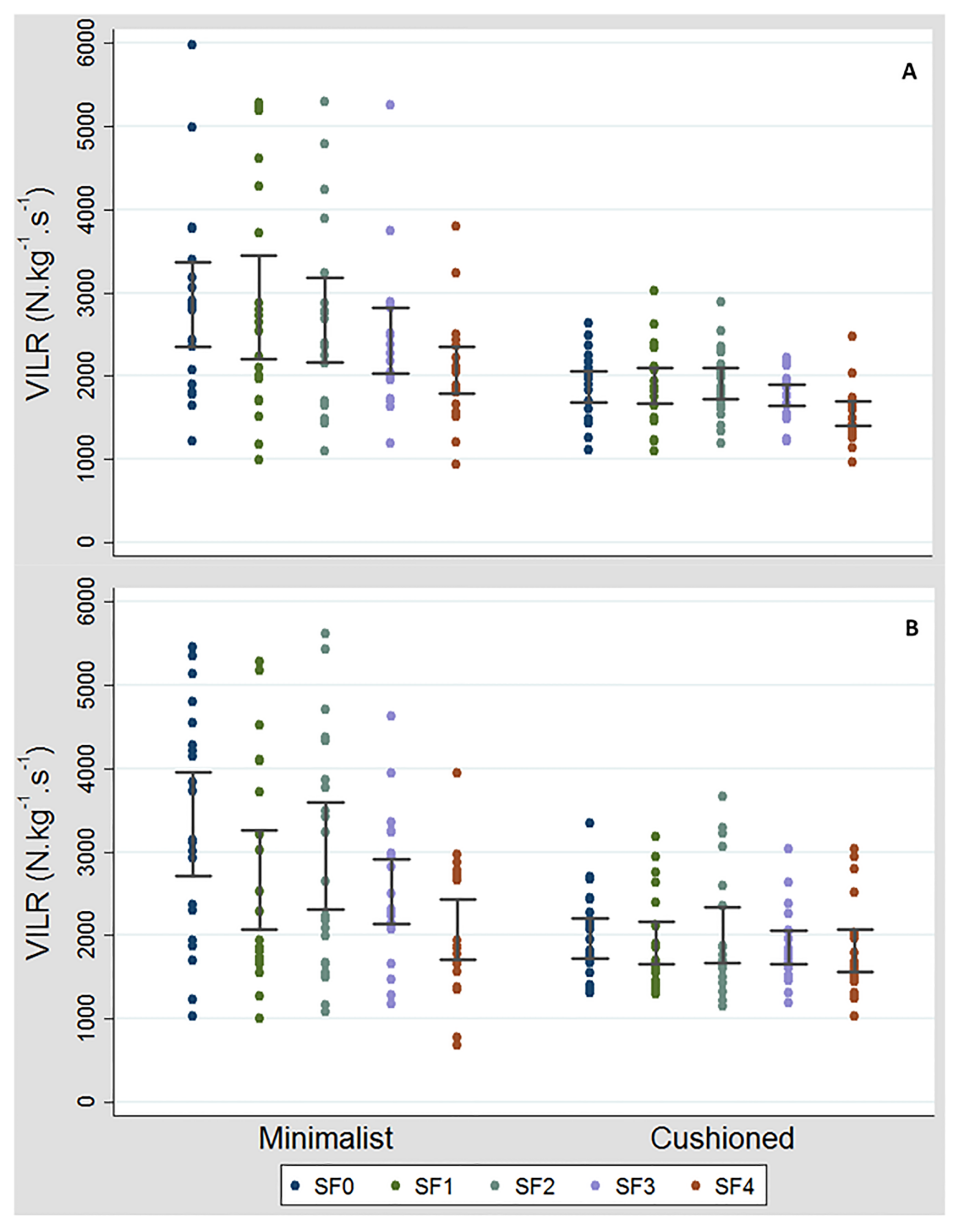
Individual results of the 21 participants for Vertical Instantaneous Loading Rate (VILR, N.kg^-1^.s^-1^) in the 10 conditions of the experiment, as well as the 95%CI of the mean, during ankle jumps (A) and multi-jumps (B).

No SF by shoe interaction was found for any variable analysed during ankle jumps. PVGRF was not significantly influenced by SF, but VILR was (ω^2^ = 0.10, Table 1). The post-hoc analysis revealed that VILR was lower in SF4 when compared to SF0 (p = 0.001, 95%CI: -939 to -174 N.s^-1^.kg^-1^), SF1 (p = 0.001; 95%CI: -929 to -165 N.s^-1^.kg^-1^) and SF2 (p = 0.004, 95%CI: -865 to -99 N.s^-1^.kg^-1^). A stratified analysis confirmed that VILR was lower in SF4 when compared to SF0 and to SF1 in MIN (see Table 2). In CUSH, VILR was lower in SF4 than in SF0, SF1, SF2 and SF3 (see also Fig 4).

**Table 1:** Impact and jump performance related variables (n = 21 participants).

Variable	Shoe type	SF0	SF1	SF2	SF3	SF4	Shoe effect (*p-value*)	SF effect (*p-value*)	Shoe*SF (p-value)
*Ankle Jumps*									
PVGRF *(N*.*kg*^*-1*^*)*	MIN	60.0±5.0	59.2±5.3	61.4±7.0	60.5±6.9	60.3±4.7	0.002	0.636	0.637
	CUSH	62.3±5.7	62.0±6.5	62.2±4.8	61.4±5.6	61.5±5.4			
VILR *(N*.*s*^*-1*^. *kg*^*-1*^*)*	MIN	2859±1117	2825±1360	2678±1113	2429±871	2074±625	<0.001	<0.001	0.414
	CUSH	1876±415	1890±472	1907±415	1772±267	1546±318			
VALR *(N*.*s*^*-1*^. *kg*^*-1*^*)*	MIN	699±132	689±124	730±188	714±137	704±115	<0.001	0.617	0.782
	CUSH	800±154	783±166	795±114	782±132	767±132			
CT *(ms)*	MIN	198±15	201±21	195±19	197±23	199±16	<0.001	0.300	0.971
	CUSH	188±18	189±18	185±14	188±17	190±13			
W_ecc_ *(J*. *kg*^*-1*^*)*	MIN	-4.0±0.5	-4.2±0.7	-4.0±0.6	-4.0±0.7	-4.0±0.6	0.008	0.524	0.972
	CUSH	-3.9±0.6	-4.0±0.7	-3.9±0.6	-3.9±0.6	-3.9±0.6			
JH *(cm)*	MIN	23.8±4.3	24.5±5.2	24.1±4.4	23.4±5.2	23.5±4.0	0.588	0.444	0.998
	CUSH	23.6±3.8	24.1±5.1	23.9± 4.4	23.4±4.4	23.4±4.2			
W_con_ *(J*. *kg*^*-1*^*)*	MIN	4.1±0.6	4.2±0.8	4.1±0.6	4.0±0.8	4.1±0.5	0.004	0.266	0.980
	CUSH	3.9±0.6	4.0±0.7	4.0±0.6	3.9±0.6	3.9±0.6			
*Multi-jumps*									
PVGRF *(N*.*kg*^*-1*^*)*	MIN	36.2±7.0	36.2±8.0	36.5±7.4	35.3±7.5	35.0±7.3	<0.001	0.240	0.740
	CUSH	31.6±5.4	32.9±6.0	34.0±7.5	32.2±5.9	32.4±6.4			
VILR *(N*.*s*^*-1*^. *kg*^*-1*^*)*	MIN	3340±1361	2666±1316	2953±1403	2523±856	2077±793	<0.001	<0.001	0.003
	CUSH	1966±541	1911±567	2001±732	1854±445	1815±565			
VALR *(N*.*s*^*-1*^. *kg*^*-1*^*)*	MIN	427±139	430±158	423±153	411±139	402±145	0.488	0.802	0.533
	CUSH	402±151	395±153	433±207	399±149	423±139			
CT *(ms)*	MIN	636±103	646±104	596±109	618±93	620±85	0.002	0.013	0.694
	CUSH	593±88	617±112	581±105	603±91	604±96			
W_ecc_ *(J*. *kg*^*-1*^*)*	MIN	-7.9±0.6	-8.0±0.7	-7.8±0.7	-8.0±0.7	-8.0±0.6	0.792	0.380	0.903
	CUSH	-7.9±0.6	-8.0±0.7	-7.9±0.7	-7.9±0.7	-7.9±0.7			
JH *(cm)*	MIN	33.4±4.8	33.6±4.0	33.4±3.7	33.9±4.9	33.2±4.3	<0.001	0.304	0.714
	CUSH	34.0±3.9	34.2±4.3	34.3±3.9	34.1±4.4	33.8±4.3			
W_con_ *(J*. *kg*^*-1*^*)*	MIN	7.8±0.8	7.9±0.7	7.7±0.6	7.9±0.7	7.8±0.6	0.061	0.171	0.900
	CUSH	7.7±0.6	7.8±0.7	7.7±0.6	7.7±0.6	7.8±0.6			

Values are Mean±SD. PVGRF: Peak Vertical Ground Reaction Force; VILR: Vertical Instantaneous Loading Rate; VALR: Vertical Average Loading Rate; CT: Contact Time; W_ecc_: Eccentric Work; JH: Jump Height; W_con_: Concentric Work; N: Newton; kg: kilogramme; s: second; ms: millisecond; cm: centimetre; J: Joule; MIN: Minimalist shoes; CUSH: Cushioned shoes; SF: Sports Floorings.

**Table 2:** Summary of significant pairwise comparisons between sports floorings for Vertical Instantaneous Loading Rate during ankle jump and multi-jump tasks after stratification according to shoe conditions (n = 21 participants).

Task	Shoe	Pairwise comparison		Mean diff.	p-value	95%CI for difference	
			(Reference)	(N.s^-1^. kg^-1^)		Lower bound	Upper bound
Ankle Jumps	MIN	SF4	SF0	-784	0.012	-1455	-112
		SF4	SF1	-750	0.018	-1422	-79
	CUSH	SF4	SF0	-329	<0.001	-545	-113
		SF4	SF1	-344	<0.001	-560	-128
		SF4	SF2	-360	<0.001	-576	-144
		SF4	SF3	-225	0.035	-441	-9
Multi-jumps	MIN	SF4	SF0	-1263	<0.001	-1948	-578
		SF4	SF2	-876	0.004	-1561	-191
		SF3	SF0	-817	0.009	-1503	-132

MIN: Minimalist shoes; CUSH: Cushioned shoes; SF: Sports Floorings; 95%CI: 95% Confidence Interval; N: Newton; s: second; kg: kilogramme.

### Effect of sports floorings on PVGR and VILR during multi-jumps

During multi-jumps, a single significant SF by shoe interaction was found for VILR (Table 1 and Fig 4). The stratified analysis revealed that in MIN, VILR was lower in SF4 compared to SF0 and SF2, and lower in SF3 compared to SF0 (Table 2). No significant difference was observed in CUSH. SF did not influence PVGRF.

### Effect of sports floorings on other impact-force-related variables

CT was the only additional variable affected by SF, but only during multi-jumps (ω^2^ = 0.05). Lower values were observed for SF2 compared to SF1 (p = 0.004, 95%CI: -0.077 to -0.009 s). After stratification, this difference was only observed in MIN (p = 0.033, 95%CI: -0.098 to -0.002 s).

### Effect of shoe conditions

Shoe conditions impacted most of the variables (Table 1). CUSH induced lower VILR during both ankle jumps (95%CI: -943 to -607 N.s^-1^.kg^-1^, ω^2^ = 0.31) and multi-jumps (95%CI: -979 to -627 N.s^-1^.kg^-1^, ω^2^ = 0.31). During ankle jumps, CUSH induced greater PVGRF (95%CI: 0.62 to 2.58 N.kg^-1^, ω^2^ = 0.05), greater VALR (95%CI: 54 to 102 N.s^-1^.kg^-1^, ω^2^ = 0.19), lower CT (95%CI: -0.013 to -0.007 s, ω^2^ = 0.17) and lower eccentric (95%CI: -0.23 to -0.04 J.kg^-1^, ω^2^ = 0.03) and concentric (95%CI: -0.26 to -0.05 J. kg^-1^; *ω*^2^ = 0.04) work compared to MIN (Table 1). During multi-jumps, PVGRF was lower (95%CI: -4.20 to -2.20 N.kg^-1^, ω^2^ = 0.18), CT was shorter (95%CI: -0.039 to -0.009 s, ω^2^ = 0.05) and JH was higher (95%CI: 0.3 to 0.9 cm; *ω*^2^ = 0.06) in CUSH compared to MIN.

## Discussion

To the authors’ knowledge, this is the first study to characterise SF regarding impact forces and jump performance during landing from standardised jump tasks using different footwear. A major finding was that VILR was lower for the softest floorings during ankle jumps, and also during multi-jumps in MIN (Table 2). In those conditions, the softest flooring presented the lowest values during both jump tasks, with the highest values observed for SF0 (control) and SF2 conditions, i.e. the hardest and the thinnest floorings (Fig 4). However, in CUSH and multi-jumps conditions, SF no longer had a significant effect on VILR. In accordance with our secondary hypothesis, overall lower VILR was observed in cushioned shoes for both jump tasks (Fig 4). These results could be relevant in the context of sport injury prevention, as overuse injuries, such as tibial stress fractures and plantar fasciitis, have previously been associated with VILR [2, 3]. Conversely, PVGRF was not attenuated by SF, which means that our primary hypothesis was only partially confirmed. Although speculative, other authors have already suggested that the rate of loading may be a better indicator of cushioning ability than impact force [20]. Since jump performance was not affected by flooring, and only marginally by footwear (mean difference: 6 mm), the effect of flooring or footwear on VILR cannot be explained by differences in JH and will be the main focus of the discussion.

The influence of cushioning properties of interfaces (i.e. footwear or SF) on impact forces and peak loading rate has been previously studied throughout a large variety of tasks. Indeed, shock absorption was investigated using impact applied under the heel [31, 32], during running at different speeds [11, 18], or during jump tasks such as drop jump from different heights [10, 13], unanticipated drop jumps [9] or single-leg step-down tasks [19]. All these tasks challenge the neuromuscular system in different manners [19], which means that factors such as postural control, lower limb angles, leg stiffness or muscle activation may influence the effect of cushioning on impact forces at landing. This could explain why impact forces and/or peak loading rate were found to be reduced [9, 28], unchanged [9, 10] or increased [11] with softer materials according to the specific task investigated.

The influence of cushioning on impact attenuation thus depends on numerous additional factors. A lower peak loading rate was observed in cushioned shoes compared to minimalist shoes during the impact phase of unanticipated drop landing, but no difference was observed during drop jumps [9]. This was confirmed by another study showing that loading rate was higher during hopping when the surface was surprisingly hard compared to a consistently soft surface, while no difference was found for loading rate between consistently hard and soft conditions [33]. Thus, cushioning could play an important role in reducing both PVGRF and VILR in unanticipated conditions.

Several studies showed an interaction between interface cushioning, knee flexion, muscle activation and limb stiffness [10, 18, 32]. In running, shoe cushioning was found to affect both limb stiffness and running kinematics [18]. Impact applied under the heel generated higher impact forces in conditions with higher muscle contraction or more extended knee, independently. Actually, both conditions independently increased limb stiffness, which should be considered as a key internal factor that influences loading rate [32]. All these observations seem to indicate that athletes can adapt limb stiffness when the underlying surface stiffness properties are modified and can be anticipated [18, 33]. This could explain why PVGRF was not modified by SF in our study. Nevertheless, PVGRF was noticeably lower in CUSH when compared to MIN during multi-jumps, which confirms our secondary hypothesis. However, the results are inconsistent between the jump tasks since PVGRF was slightly higher in CUSH during ankle jumps. Also, the effect size is much lower than for VILR. A previous study showed that PVGRF was not different in the shod condition when compared to barefoot during double-leg drop landings from 0.3 and 0.6 m heights [10]. Indeed, athletes seem to adapt in contrasting ways to different conditions according to the task.

A shorter CT was observed in SF2, the thinnest flooring, when compared to SF1 during multi-jumps. Interestingly, a previous study showed that CT was shorter when hoppers landed on an expected hard surface compared to a consistently soft surface [33]. Thus, we could speculate that the participants anticipated a hard surface because of the limited thickness of the flooring and adapted their technique accordingly. However, the effect is small, no difference was observed between the other floorings, and this observation is not supported by the comparison between MIN and CUSH shoes where a shorter CT was found in cushioned shoes during both ankle jumps and multi-jumps.

According to our secondary hypothesis, many biomechanical variables were influenced by shoe conditions, and therefore, most probably by shoe cushioning properties. Some effects were common to both jump tasks (e.g. VILR), while others were task-specific. For example, PVGRF was considerably lower in CUSH during multi-jumps, while it was slightly higher during ankle jumps. The latter observation confirms that investigating the influence of shock absorption properties of sports equipment (e.g. shoes, floorings…) on human biomechanics in different jump tasks is relevant and might provide complementary information.

To the best of our knowledge, our study is the first to analyse the relationship between SF and jump performance. In addition, studies relating footwear and jump performance are scarce. In a group of recreational athletes with experience in jump task, height during a vertical jump task was lower in tennis shoes when compared with barefoot and minimalist footwear conditions.[17] However, this difference was observed in men only. Another study found no difference in vertical jump performance between barefoot, minimalist and conventional running shoes.[21] In the present study, with the exception of a slightly better jump performance (6 mm) in CUSH for the multi-jumps task, no influence of footwear and SF conditions on jump height was found. Within the experimental conditions analysed to date, it seems that shock attenuation properties of sports equipment has minimal impact on jump performance.

Our study design was robust as the order of the conditions was randomised. Furthermore, both flooring and shoe cushioning properties were investigated. Finally, the researchers were blinded as to the SF characteristics until the end of the data treatment and thus less prone to be biased. One limitation of this study is that the influence of floorings on shock attenuation was not investigated at different jump heights. Also, our analysis was restricted to force measurements. Adding other measurement techniques, like motion analysis or electromyography, might have provided more insight on biomechanical adaptations to SF cushioning properties. Finally, shoe conditions differed in many aspects such as stack height at the heel, heel-toe drop, and weight (Fig 2). Thus, any shoe effect could not be attributed to cushioning properties alone.

## Conclusion

Cushioning influences impact forces during standardised jump tasks, whether it is provided by the shoes or the SF. VILR is the variable that was the most often affected by both shoe and SF conditions. This is clinically interesting given that VILR was previously associated with sports injury risk. Surprisingly, many variables were affected by the shoe type but not by the SF. Further study is needed to understand biomechanical adaptation to SF cushioning, as well as to identify the best solution to lower VILR without affecting jump performance.

## Supporting information

S1 File Dataset. (CSV)
